# Management and Prognosis of Sustained Ventricular Tachycardia in the Emergency Department: A Retrospective, Observational, Single-Centre Study

**DOI:** 10.3390/jcm15207805

**Published:** 2026-10-10

**Authors:** Francesco Salinaro, Pietro Begnini, Roberto Rordorf, Santi Di Pietro, Benedetta Traversi, Debora Monti, Andrea Simone Dedato, Bruno Barcella, Enrico Moi, Maya Coridori, Enrico Baldi, Simone Savastano, Rita Camporotondo, Stefania Guida, Leonardo De Luca, Stefano Perlini

**Affiliations:** 1 Emergency Medicine Unit and Emergency Medicine Postgraduate Training Program, Department of Internal Medicine, University of Pavia, IRCCS Policlinico San Matteo Foundation, 27100 Pavia, Italy; pietro.begnini@gmail.com (P.B.); santi.dipietro@unipv.it (S.D.P.); benedetta.traversi01@universitadipavia.it (B.T.); debora.monti01@universitadipavia.it (D.M.); andreasimone.dedato01@universitadipavia.it (A.S.D.); bruno.barcella.92@gmail.com (B.B.); enrico.moi01@universitadipavia.it (E.M.); stefano.perlini@unipv.it (S.P.); 2 Division of Cardiology, IRCCS Policlinico San Matteo Foundation, 27100 Pavia, Italy; r.rordorf@smatteo.pv.it (R.R.); enrico.baldi@unipv.it (E.B.); s.savastano@smatteo.pv.it (S.S.); r.camporotondo@smatteo.pv.it (R.C.); s.guida@smatteo.pv.it (S.G.); l.deluca@smatteo.pv.it (L.D.L.); 3 Division of Paediatric Cardiology, IRCCS Policlinico San Matteo Foundation, 27100 Pavia, Italy; maya.coridori03@gmail.com

**Keywords:** ventricular tachycardia, management, prognosis, ECG

## Abstract

**Background/Objectives:** Sustained ventricular tachycardia (VT) is a potentially life-threatening arrhythmia requiring prompt Emergency Department (ED) assessment and management. However, data on its frequency, real-world treatment, and prognosis in the ED remain limited. We aimed to describe the clinical characteristics, acute management, and short- and long-term outcomes of patients presenting with VT. **Methods:** We retrospectively included consecutive patients presenting to the ED between January 2018 and December 2024 with a primary diagnosis of sustained VT with a pulse. We collected baseline characteristics, hemodynamic status, underlying cardiac disease, and acute treatment. In-hospital events and 30-day and 1-year outcomes were assessed. **Results:** A total of 103 patients were identified, accounting for approximately 0.025% of all ED visits during the study period; 84.5% were male. Chest pain (31.1%) and palpitations (25.2%) were the most common presenting symptoms. Structural heart disease was present in 90% of the cases, predominantly of ischemic origin (61%). Nearly half of the patients (49.5%) had a prior history of VT. Electrical cardioversion was performed in 28.2% of the cases. The majority of the patients (95/103; 92%) required hospitalization. Mortality was 4.9% at 30 days and 17% at 1 year. After adjustment for age, a left ventricular ejection fraction ≤ 30% and in-hospital arrhythmic recurrence were independently associated with 1-year mortality. **Conclusions:** Sustained VT is an uncommon but high-risk ED presentation, mainly affecting patients with ischemic structural heart disease. A discrepancy was observed between real-world clinical practice and guideline recommendations. The substantial mortality observed, particularly among patients with impaired left ventricular function or in-hospital arrhythmic recurrence, highlights the need for prompt risk stratification and optimized, guideline-concordant acute management.

## 1. Introduction

Ventricular tachycardia (VT) represents the most common cause of wide-complex tachycardia. Sustained ventricular tachycardia is defined as a ventricular arrhythmia lasting more than 30 s or leading to hemodynamic compromise within 30 s and requiring intervention. These arrhythmias can arise from, or involve, specialized conducting tissue as well as ventricular myocardium. VT can occur in structurally normal or abnormal hearts and can be hemodynamically tolerated or not. Ischemic heart disease is the most common cause of ventricular tachycardia, and VT has a wide range of clinical presentations, including palpitations, chest pain, shortness of breath, syncope, and cardiac arrest [1,2,3,4]. The 2022 European Society of Cardiology (ESC) Guidelines emphasize a structured approach to ventricular arrhythmias, integrating prompt treatment of the acute episode with identification of the underlying substrate and individualized risk stratification for sudden cardiac death. In patients with sustained VT, immediate synchronized electrical cardioversion is recommended in the presence of hemodynamic instability, while electrical cardioversion or intravenous antiarrhythmic therapy may be considered in hemodynamically tolerated monomorphic VT, according to the clinical context and the safety of procedural sedation. Management should also include the identification and correction of reversible triggers, as well as early consideration of catheter ablation and implantable cardioverter–defibrillator therapy in selected high-risk patients [4]. Furthermore, accumulating evidence suggests that stellate ganglion block may represent a valuable adjunctive or bridging intervention in patients with refractory ventricular arrhythmias and electrical storm [5,6]. Nevertheless, in real-world settings, the immediate availability of invasive procedures and of the personnel, expertise, and resources required to safely deliver procedural sedation and analgesia may affect treatment selection and adherence to guideline-recommended care.

However, information on clinical characteristics, acute management and outcomes of patients with sustained VT in an emergency department (ED) is still limited.

The aim of the present study was to provide a detailed description of clinical characteristics, presentation, treatment and prognosis of patients who were admitted to the emergency department of a tertiary care hospital for sustained VT.

## 2. Methods

### 2.1. Study Design and Population

This is a single-center retrospective study including consecutive patients with sustained VT presenting to the ED of the Fondazione IRCCS Policlinico San Matteo of Pavia, which handles approximately 5000 patients per month. We reviewed the medical records of patients who presented with sustained VT (ICD-9-CM diagnostic code 427.1, ‘Paroxysmal ventricular tachycardia’) and were treated in this ED between January 2018 and December 2024. All potentially eligible cases identified through ICD-9-CM code 427.1 underwent manual review of the electronic medical record, including available 12-lead ECG recordings, clinical documentation, and ICD interrogation reports. Only patients with confirmed sustained VT with a pulse were included in the final analysis. Patients with wide QRS supraventricular tachycardia, non-sustained VT and those with pulseless VT were excluded.

### 2.2. Clinical Management and Follow-Up

All patients had their medical history recorded and underwent physical examination, 12-lead ECG recording, routine laboratory testing, and chest X-ray. For those patients presenting with ongoing VT, the diagnosis was based on clinical history and current standard 12-lead ECG criteria [7,8,9,10], including: absence of an RS complex in all precordial leads; an R–S interval > 100 ms in any precordial lead; presence of atrioventricular dissociation; fusion or capture beats; extreme axis deviation (from −90° to +180°); and morphological criteria for VT in the precordial leads. In patients presenting to the ED with an implanted cardioverter-defibrillator (ICD), the diagnosis of appropriate VT therapy was confirmed by ICD interrogation and retrieval of the clinical episode. Patients were considered stable if they had stable vital signs, including systolic blood pressure > 90 mmHg and absence of signs of end-organ hypoperfusion.

Information on ED management was collected by reviewing patients’ ED records. Data on whether patients were discharged or admitted to the hospital, and to which unit (Cardiology ward, Intensive Care Unit, General Intensive Care, or other), were also collected. Clinical outcomes and treatments received until hospital discharge were retrieved from patients’ medical records. Moreover, follow-up information at 30 days and 1 year was obtained by reviewing re-access and death registries or through telephone contact with patients or caregivers to assess clinical status.

### 2.3. Endpoint Definitions

Beyond describing clinical management during the ED stay, our objective was to portray patient outcomes during the subsequent hospitalization. To achieve this aim, we defined two in-hospital endpoints to evaluate the prognosis of our cohort: (1) a **clinical endpoint**, defined as a composite of in-hospital mortality, need for vasoactive drugs, and/or intra-aortic balloon pump; and (2) an **arrhythmic endpoint**, defined as the need for antiarrhythmic drugs, ICD interventions, stellate ganglion block, electrical cardioversion, or external defibrillation for relapsing ventricular arrhythmias occurring after the index episode and beyond the ED stay.

### 2.4. Statistical Analysis

Categorical variables were expressed as absolute numbers and percentages. Continuous variables were reported as median, minimum, and maximum values. Normality of distribution for continuous variables was initially assessed using histograms and the Shapiro–Wilk test. In cases of normally distributed data, Bartlett’s test was applied to assess homogeneity of variances. When both normal distribution and homogeneity of variances were confirmed, a *t*-test was then performed. Conversely, in the presence of abnormal distribution and/or heterogeneity of variances, the Wilcoxon rank-sum test was used. For categorical variables, either the Chi-square test or Fisher’s test was applied, depending on sample size.

Results were considered statistically significant at a *p*-value < 0.05. For parameters reaching statistical significance, a logistic regression model was performed, and *p*-values and odds ratios (OR) were reported. A preliminary bivariate analysis was conducted on key variables, adjusting for age, in order to identify potential 1-year mortality associations. Based on these findings, a multivariate logistic regression model was built. Missing data were imputed using the median for continuous variables and the mode for categorical variables. Missing data accounted for less than 5% of observations across all variables included in the database and for less than 3% of observations for the variables entered into the regression model. Survival analysis was performed using Kaplan–Meier curves for the overall population and stratified by the recurrence of arrhythmic events. For patients who were lost to follow-up, the date of last medical contact was considered. Statistical analysis was performed using R version 4.4.2.

## 3. Results

### 3.1. Clinical Endpoints

During the study period, nearly 400,000 patients were admitted to the ED. Of these, 103 showed sustained ventricular tachycardia (VT), accounting for approximately 0.025% of all visits. The final study population included 103 patients, of whom 87 were male (84.4%) and 16 were female (15.5%). The median age at ED admission was 71 years (range 24–96), and the median body mass index (BMI) was 26 kg/m^2^.

**Figure 1:**
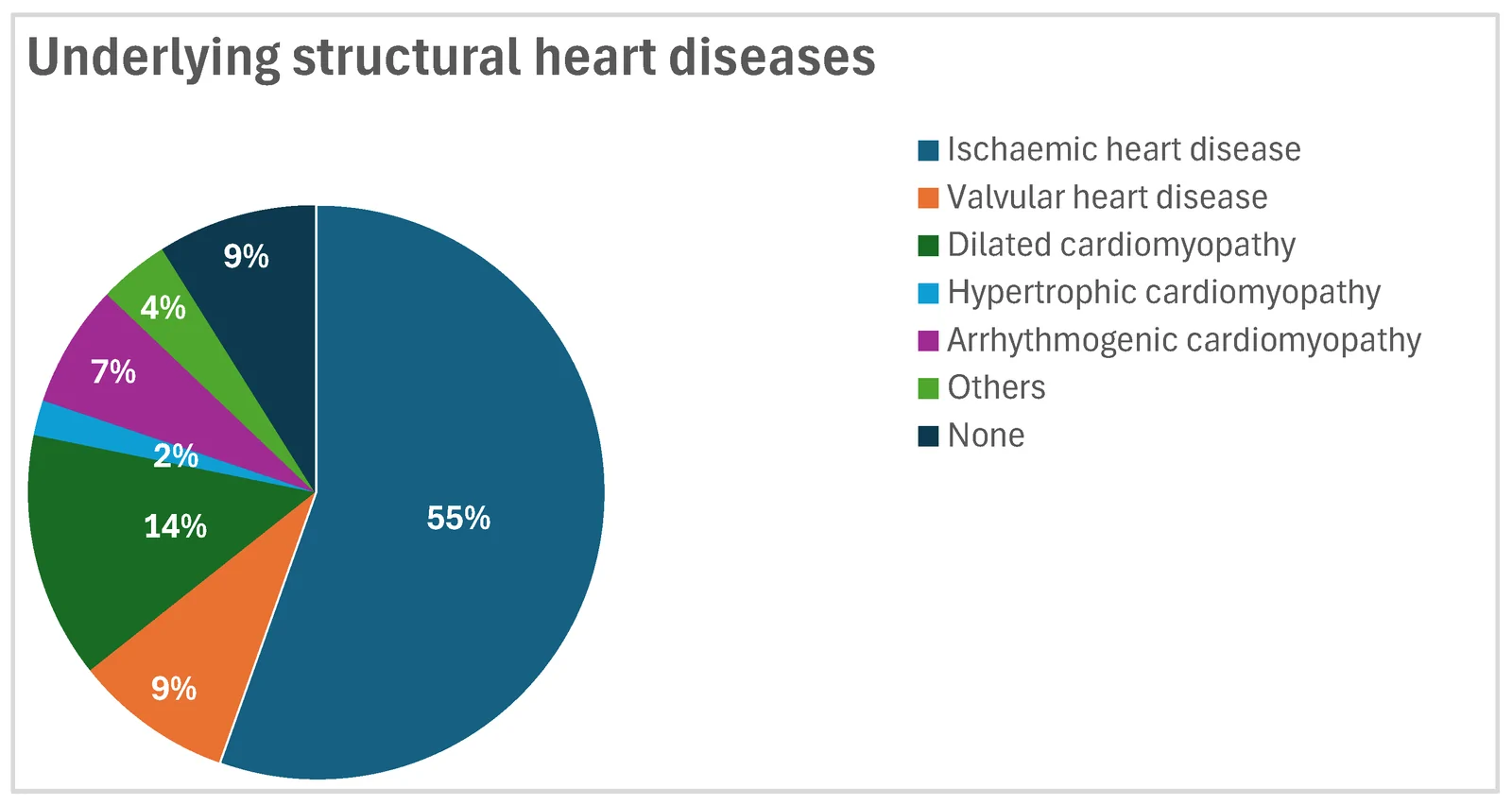
Distribution of underlying heart disease in the study population.

Table 1 summarizes the relevant medical history and clinical characteristics of the enrolled patients. As shown in Figure 1, 90% of patients had a previously diagnosed structural heart disease, predominantly of ischemic etiology (61%). Specifically, six patients presented with acute or subacute myocardial infarction, while 50 had a history of chronic ischemic heart disease without acute events. Dilated non-ischemic cardiomyopathy was observed in 15.2% of patients, valvular heart disease in 9.8%, arrhythmogenic cardiomyopathy in 7.6%, and hypertrophic cardiomyopathy in 2.2%. Additionally, one patient had a dilated hypokinetic cardiomyopathy with arrhythmogenic features not yet fully characterized; one had dilated hypokinetic cardiomyopathy associated with Emery–Dreifuss syndrome; one had dilated cardiomyopathy with non-compaction features; and one had cardiac sarcoidosis.

**Figure 2:**
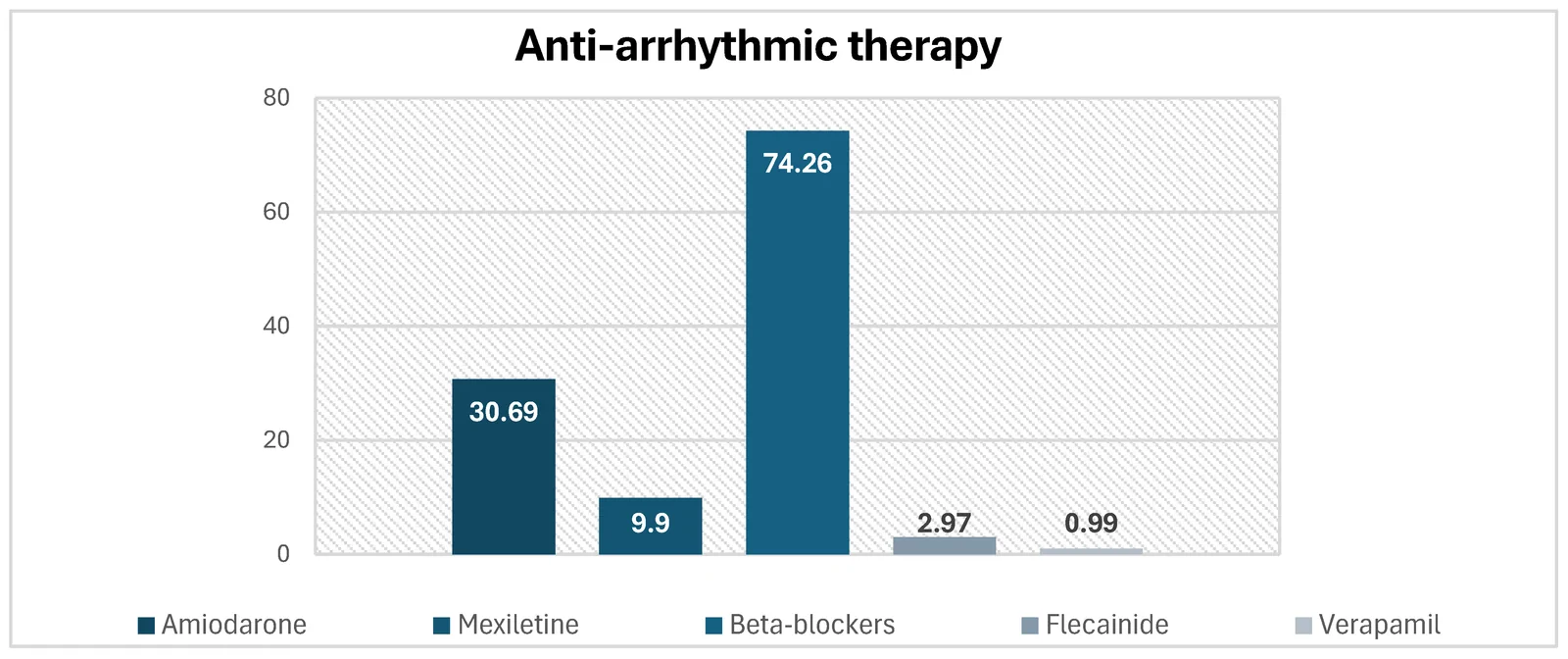
Distribution of chronic antiarrhythmic therapy at baseline in patients presenting with sustained ventricular tachycardia. At presentation, mild hypokalemia (serum potassium < 3.5 mmol/L) was observed in 12 patients, whereas hyperkalemia (serum potassium > 5.0 mmol/L) was present in 3 patients. Serum creatinine was >1.2 mg/dL in 48.5% of patients. The median peak high-sensitivity cardiac troponin I (hs-cTnI) concentration was 124 ng/L (range, 0–61,894 ng/L); 53.8% of patients had values above the sex-specific upper reference limit (37 ng/L in women and 57 ng/L in men). Sixty-five patients presented with ongoing ventricular tachycardia at the time of medical evaluation, while the others were diagnosed based on the ICD interrogation. Within the subgroup of 65 patients with sustained VT during ED evaluation, 21 (32%) were hemodynamically unstable, with systolic blood pressure (SBP) < 90 mmHg).

Among patients with an available standard 12-lead ECG documenting the index arrhythmic episode, monomorphic VT was substantially more common than polymorphic VT. The most recent pre-admission assessment of left ventricular ejection fraction (LVEF) indicated marked impairment of left ventricular systolic function, with a median LVEF of 35%. Only 15% of patients had a previously documented LVEF ≥ 50%. Regarding arrhythmic history, the index ED presentation represented a recurrence of VT in 49.5% of patients. Previous catheter ablation for VT had been performed in 18.1% of patients, whereas cardiac sympathetic denervation had been performed in 2.0%. Moreover, 63.7% of the patients had an implantable cardioverter-defibrillator (ICD). Among ICD carriers, 44.6% had a single-chamber device, 10.8% a dual-chamber device, 41.5% a cardiac resynchronization therapy defibrillator (CRT-D), and 3.1% a subcutaneous ICD (S-ICD). Chronic antiarrhythmic therapy was prescribed in 85 patients; treatment regimens are summarized in Figure 2.

### 3.2. Acute Management in the ED

**Figure 3:**
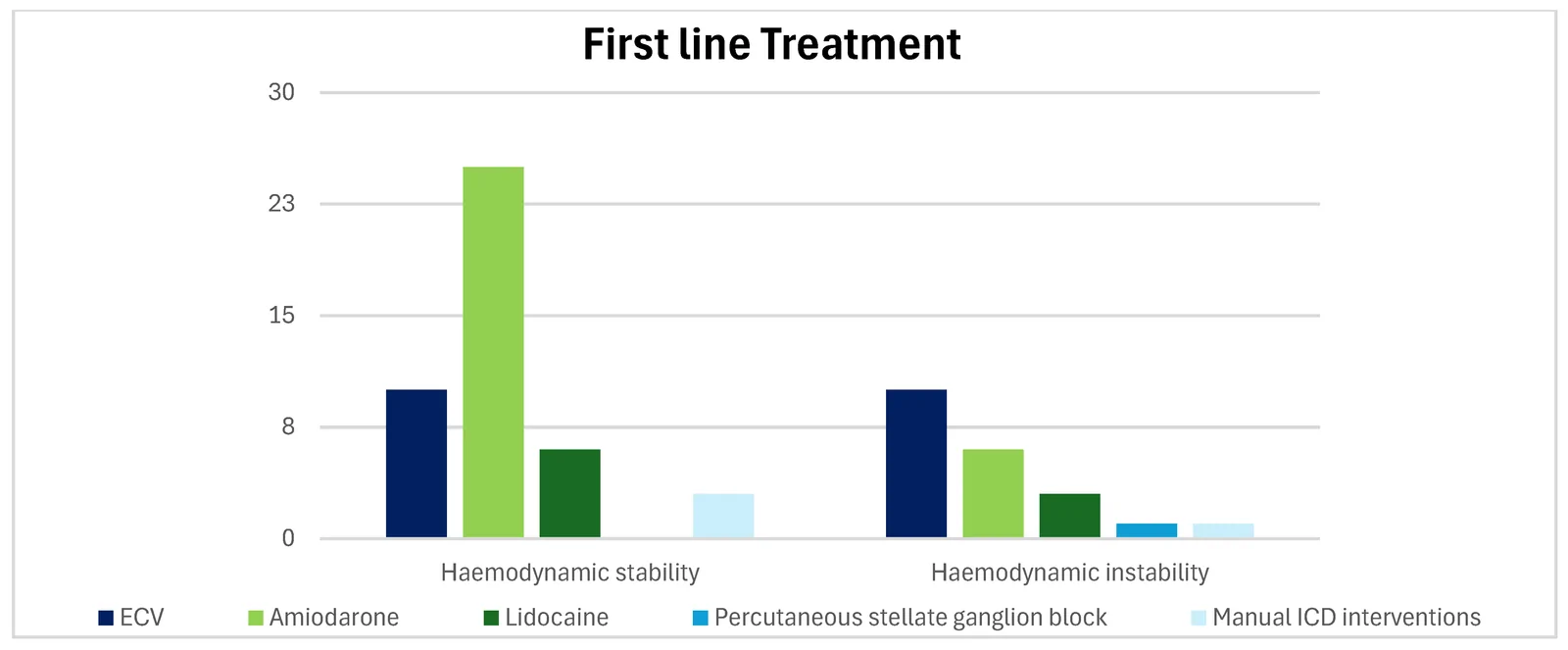
Distribution of first-line antiarrhythmic treatment used for sustained ventricular tachycardia, according to hemodynamic presentation. ECV, electrical cardioversion.

Among the 65 patients presenting with ongoing sustained VT, 44 were hemodynamically stable and 21 were hemodynamically unstable. First-line treatment according to hemodynamic status is shown in Figure 3. Pharmacological therapy, predominantly amiodarone, was the most frequently selected initial strategy in hemodynamically stable patients, whereas electrical cardioversion (ECV) was used most often in hemodynamically unstable patients.

In the subgroup of hemodynamically stable patients, 25 were first treated with amiodarone, six with lidocaine, 10 underwent electrical cardioversion (ECV), and three were managed with manual overdrive pacing and/or ICD reprogramming.

Among hemodynamically unstable patients, ECV was the most frequently used first-line treatment, performed in 10 patients (less than 50%). Six patients initially received amiodarone, and three received lidocaine. One patient underwent percutaneous stellate ganglion block (SGB) as the initial intervention, and one was treated with manual overdrive pacing and/or ICD reprogramming.

In the overall cohort, all treatments administered in the ED were recorded, including sequential therapies required to achieve arrhythmia termination. Treatment details are summarized in Table 2. Amiodarone (50.5%) and lidocaine (19.4%) were the most frequently used antiarrhythmic agents. No patient received procainamide or verapamil in the ED. Procainamide was administered only after hospital admission in four patients managed in the coronary intensive care unit. Electrical cardioversion was performed in 20 subjects; specifically, in 69% of these cases, it was chosen as first-line therapy. Finally, eight patients underwent percutaneous stellate ganglion blockade during the hospital stay. Among patients with an ICD, 68.7% had documented automatic or manual ICD interventions.

### 3.3. Clinical Course During Hospitalization

**Figure 4:**
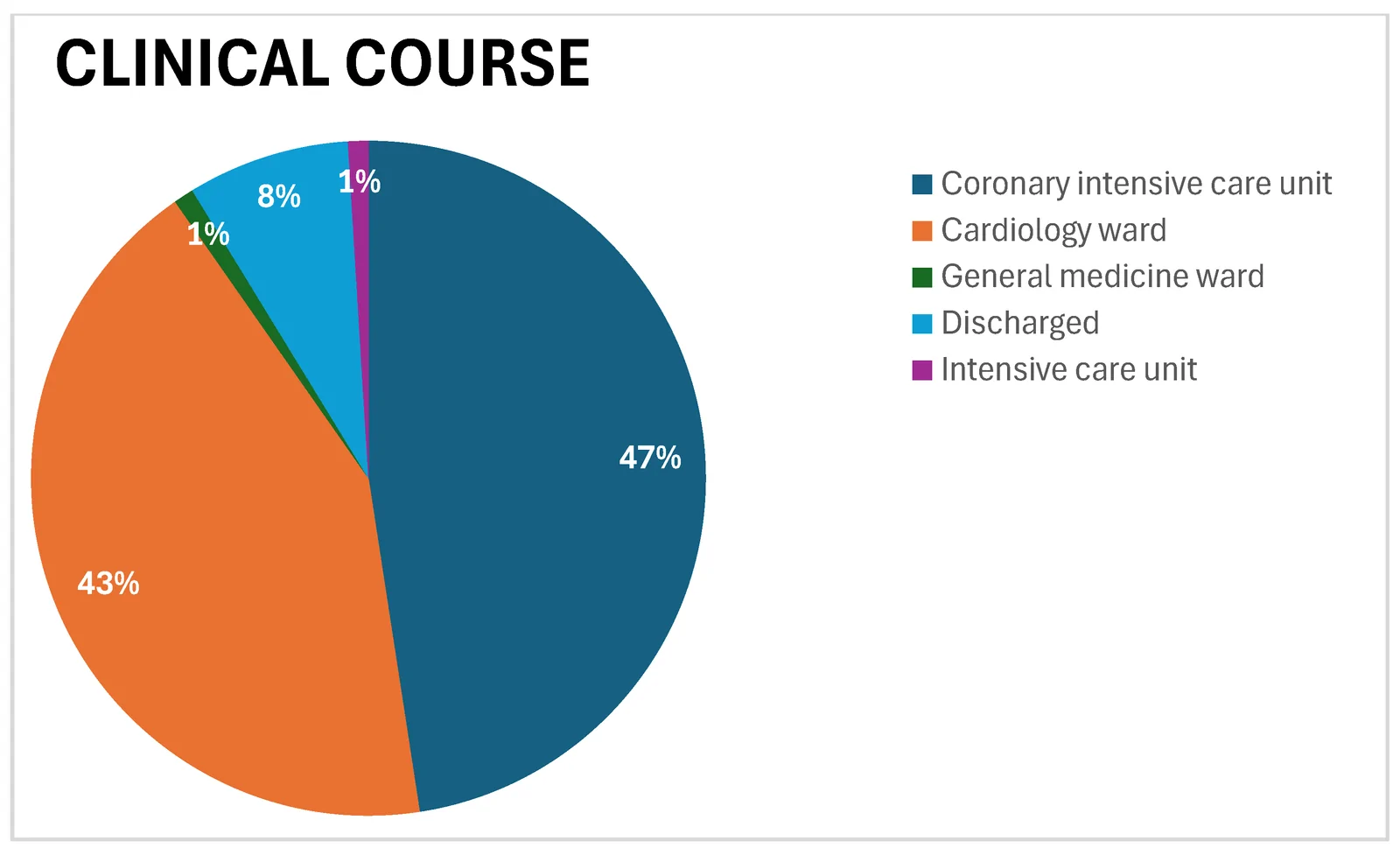
Distribution of hospital admission units: 49 patients in the CICU, 44 patients in the Cardiology ward, 1 patient in the ICU, 1 patient in the General Medicine ward, and 8 subjects discharged.

Ninety-two percent of the patients were hospitalized, mainly in the coronary intensive care unit (Figure 4). The median length of stay was 10 days, with a minimum of 3 days and a maximum of 81 days. During hospitalization, 25.8% of patients underwent electrophysiological study, and approximately 75% of them received ablation of the arrhythmic substrate. Nine patients received an ICD: three were implanted with a single-chamber ICD, three with a subcutaneous ICD, two with a dual-chamber ICD, and one patient received a CRT-D. Four patients underwent cardiac denervation, while coronary angiography was performed in 32.3% of the patients, of whom 33% required percutaneous coronary intervention.

During the hospital stay, 27 patients (26,2%) experienced one of the clinical endpoints as previously defined. Specifically, two patients died during hospitalization, 11 patients needed vasoactive drugs and/or an intra-aortic balloon pump. Furthermore, 36 patients (34.9%) experienced at least one of the arrhythmic endpoints as previously described. More specifically, 51 patients required additional intravenous antiarrhythmic therapy, 14 experienced ICD interventions, four underwent stellate ganglion block, and eight required electrical cardioversion or external defibrillation.

### 3.4. Clinical Prognosis

Two patients (1.9%) died during hospitalization. After discharge, 30-day mortality was 4.9% and 1-year mortality reached 17.2%. Patients deceased within one year were older (median age was 74 vs. 70 years, *p* < 0.05). Patients with idiopathic VT had a better prognosis when compared with those with structural heart disease, in terms of both 1-year mortality (11% vs. 17%; *p* < 0.05), reaching the clinical endpoint (20% vs. 27%; *p* < 0.05), and the arrhythmic endpoint (30% vs. 35%; *p* < 0.05).

**Figure 5:**
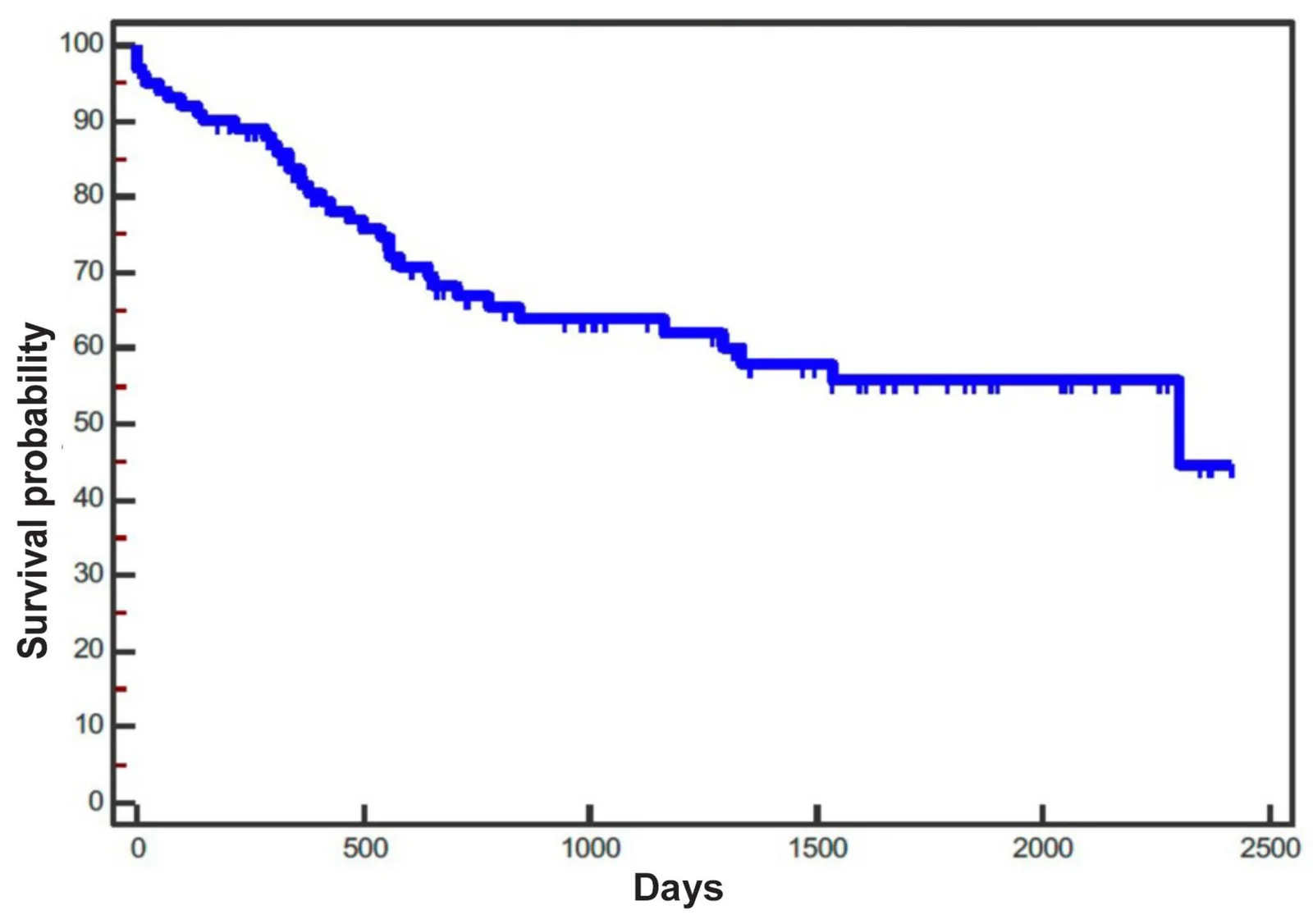

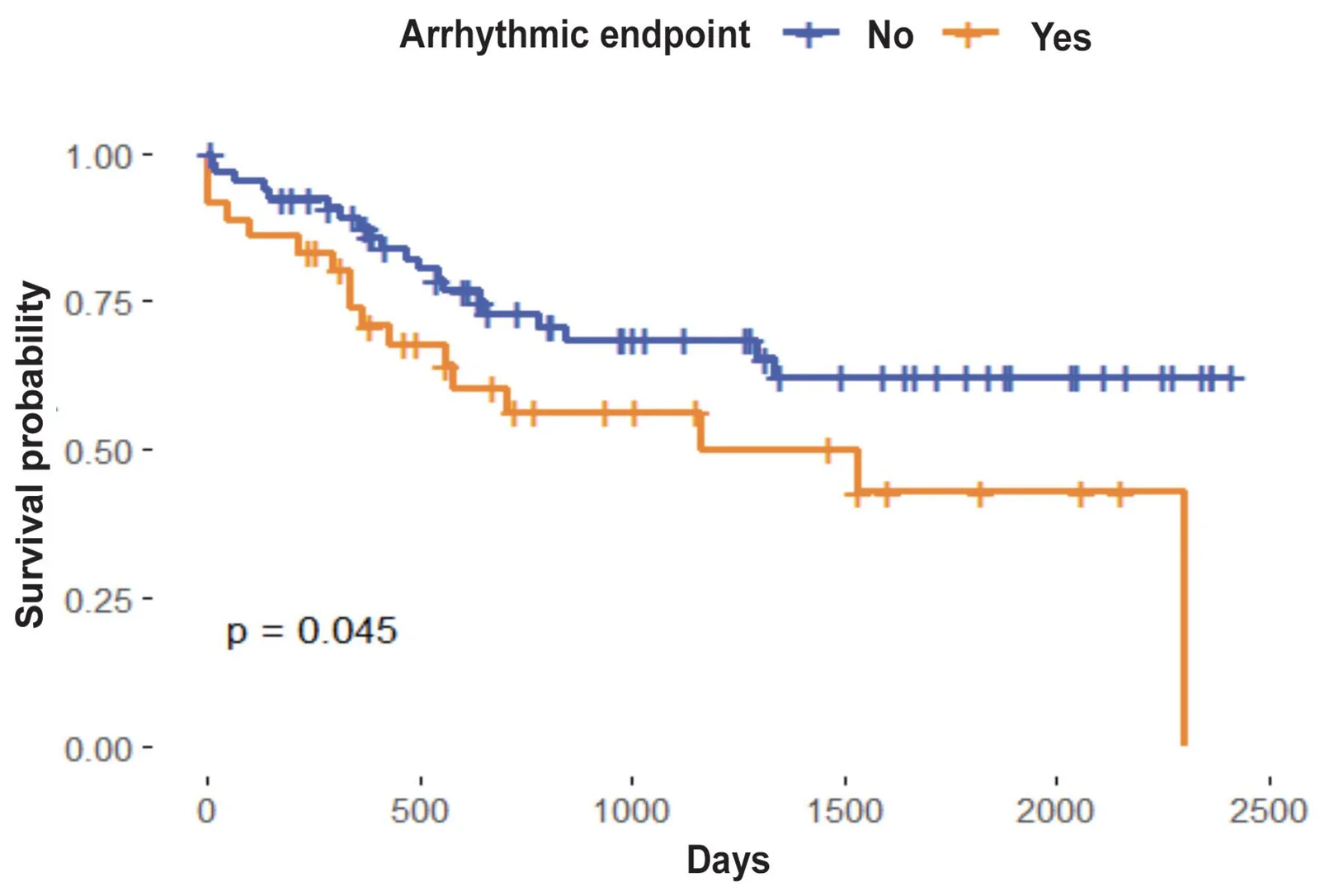
Kaplan–Meier curve for the whole study population and for patients with and without arrhythmic recurrence during hospital stay.

As reported in Figure 5, Kaplan–Meier analysis showed a 64% survival at 1000 days after discharge, decreasing to 45% at 2400 days.

Ejection fraction ≤ 30%, even after adjusting for age, was strongly associated with 1-year mortality (OR 10.158; *p* = 0.003) as well as age-adjusted serum creatinine (OR 2.45; *p* = 0.048).

Patients who experienced arrhythmic recurrence during hospitalization had a 7.42-fold higher risk of death (*p* = 0.028) within 1 year after discharge compared with those without in-hospital arrhythmic recurrences. In multivariable analysis, arrhythmic recurrence during hospitalization showed the strongest association with 1-year mortality, regardless of a prior history of cardiomyopathy. Indeed, the distribution of ischemic cardiomyopathy and other structural heart diseases reflected that of the overall study population (91% with structural cardiomyopathy, 59% of whom had an ischemic etiology). To reduce the risk of overfitting, we also revised the primary multivariable analysis by restricting the model to a smaller number of clinically prespecified covariates. Specifically, we tested Model A: in-hospital arrhythmic recurrence and age, and Model B: in-hospital arrhythmic recurrence and LVEF ≤ 30%. In Model A, arrhythmic recurrence remained better associated with one-year mortality (OR 3.925; *p* = 0.020). Similarly, in Model B, arrhythmic recurrence remained superior, associated with one-year mortality (OR 4.20; *p* = 0.028).

## 4. Discussion

The present study provides three main findings. First, ischemic heart disease remained the predominant substrate for sustained ventricular tachycardia (VT). Second, acute management in the Emergency Department (ED) frequently diverged from contemporary guideline-recommended pathways. Third, advanced age, severely impaired left ventricular systolic function (LVEF < 30%), elevated serum creatinine, and in-hospital arrhythmic recurrence were independently associated with an increased risk of one-year mortality. Notably, arrhythmic recurrence during the index hospitalization was the most powerful predictor in the multivariable analysis.

Although limited in absolute terms, our sample size compares favorably with those of several previously published single-center studies of sustained VT, supporting the clinical relevance of this real-world ED cohort [7,8,9]. Our cohort (n = 103) consisted predominantly of elderly, male, and overweight patients, with structural heart disease documented in approximately 90% of cases, most commonly of ischemic etiology. Compared with earlier series, our patients were older and more frequently male, a difference that may reflect demographic changes, improved survival after cardiovascular events, and the increasing complexity of patients presenting to the ED. Idiopathic VT was observed more frequently than in previous reports and, as expected, was associated with more favorable outcomes, consistent with the available literature [4,10,11,12]. The prevalence of acute myocardial infarction (AMI) was substantially lower than that reported by Domanovits et al. [7], in whom AMI was diagnosed in nearly one-third of VT presentations. This discrepancy may be partly explained by our case-identification strategy, which relied on the ICD-9-CM code for paroxysmal VT and may therefore have excluded patients whose primary discharge diagnosis was acute coronary syndrome. Because patient identification relied solely on ICD-9-CM code 427.1, our study may have underestimated the true number of sustained VT presentations by excluding cases coded primarily as acute myocardial infarction or cardiac arrest. This selection strategy limits generalizability to the full spectrum of VT presentations but focuses the analysis on patients in whom sustained VT represented the main acute clinical problem and for whom antiarrhythmic management and rhythm-control strategies were central to ED decision-making. This selection bias may have partially contributed to the more favorable prognosis observed in our cohort.

Median LVEF values (35% historically, 30% during hospitalization) confirmed a population with markedly impaired cardiac function. A substantial proportion of patients had an implantable cardioverter-defibrillator (ICD), often presenting due to device shocks or arrhythmic recurrences, while prior catheter ablation was uncommon. Chest pain was the most frequently reported symptom, followed by palpitations, consistent with the study conducted by Havakuk et al. [12] and descriptions in major internal medicine and cardiology textbooks [13].

Acute treatment patterns revealed a clinically meaningful gap between evidence and practice. Adenosine was administered in nine patients with regular monomorphic, hemodynamically stable VT, in accordance with ACLS guidelines, to evaluate wide-complex tachycardia of uncertain origin and exclude supraventricular tachycardia [4,14,15]. In patients with hemodynamically tolerated VT, pharmacological therapy—most commonly amiodarone—represented the predominant first-line strategy. This may reflect its widespread availability, familiarity among emergency clinicians, and perceived short-term safety. However, intravenous procainamide was not used in the ED and was administered only rarely during hospitalization. Despite growing evidence supporting procainamide as the most effective antiarrhythmic for stable monomorphic VT, continued use of lidocaine further suggests a discrepancy between contemporary evidence and real-world prescribing patterns [16,17,18,19,20]. Studies evaluating procainamide, lidocaine, amiodarone and sotalol are still limited and nevertheless, the strength of the evidence base remains vitiated by the small number of trials, heterogeneity in patient populations and eligibility criteria, and differences in outcome definitions [16,17,18,19,20]. The absence of procainamide use in the ED may have reflected not only clinical inertia or greater familiarity with amiodarone, but also practical factors such as its inclusion in the standard ED formulary and the availability of staff specifically trained in its administration and monitoring. In spite of guideline recommendations, even electrical cardioversion (ECV) was underutilized in stable patients and not consistently applied as first-line therapy in unstable cases, where it remains the recommended strategy. Although anesthetic risk may have influenced decision-making, pharmacological treatment should not delay electrical cardioversion in unstable patients and may itself cause hypotension or arrhythmic events. However, this result should be interpreted in light of the study period, as more than half of patients were enrolled before publication of the most recent ESC guideline update in 2022 [4].

Beyond individual clinician preference, organizational factors may contribute to this evidence–practice gap. Although electrical cardioversion is an established and highly effective intervention, its prompt delivery may depend on the immediate availability of trained staff, appropriate monitoring, resuscitation equipment, and procedural sedation and analgesia. Even ED overcrowding can play a role. In real-world ED practice, limitations in these resources may influence treatment selection and reduce adherence to guideline-recommended pathways. The increasing availability of emergency physicians with formal training and experience in procedural sedation and invasive bedside procedures may facilitate wider implementation of ECV. Concurrently, the use of regional anesthesia and peripheral nerve blocks in emergency medicine is expanding beyond analgesia for multiple rib fractures, with growing application across a range of procedures [5,21]. This evolving expertise may also support the future integration of autonomic modulation techniques, including stellate ganglion block, as rescue or bridging strategies in selected patients with refractory ventricular arrhythmias or electrical storm. Notably, eight patients in our cohort underwent stellate ganglion block during hospitalization: the procedure was used early in the treatment course in one patient and as rescue therapy in the remaining seven patients. Although our study was not designed to assess its efficacy, this experience reflects the increasing incorporation of stellate ganglion block into the multidisciplinary management of highly selected patients with refractory ventricular arrhythmias.

Prognostic analysis confirmed the central role of baseline cardiac and systemic disease severity. An LVEF ≤ 30% was strongly associated with one-year mortality, conferring a more than ninefold increase in the risk of death. This result is consistent with previous studies identifying severe left ventricular dysfunction as a major prognostic marker and as a key criterion for primary-prevention ICD implantation in patients at risk of ventricular tachyarrhythmias. Elevated serum creatinine was also moderately associated with mortality, likely reflecting the contribution of renal dysfunction, cardiorenal disease, and overall clinical complexity. Most importantly, in-hospital VT recurrence was associated with a 6.4-fold increase in one-year mortality. This observation identifies early arrhythmic instability as a clinically relevant marker of adverse prognosis and supports the need for prompt recognition of high-risk patients, early optimization of antiarrhythmic and hemodynamic management, and timely referral for advanced electrophysiological assessment when appropriate.

## 5. Limitations

This study has several limitations. First, it was conducted retrospectively in a single Emergency Department of a tertiary-care IRCCS Hospital. This may limit the generalizability of our findings. Our cohort likely reflects a referral population with greater baseline cardiac complexity, including a higher burden of structural heart disease, severe left ventricular dysfunction, ICD carriers, and recurrent ventricular arrhythmias than would be expected in community EDs. Furthermore, the design may have introduced selection bias, particularly in the identification of VT cases based on administrative coding ICD-9. Finally, the cause of death was not systematically recorded, limiting further analysis of mortality outcomes.

## 6. Conclusions

In this real-world ED cohort, sustained VT with a pulse was an uncommon but high-risk presentation, occurring predominantly in patients with structural heart disease of ischemic origin. Acute management frequently diverged from guideline-recommended care. One-year mortality was substantial and was independently associated with severely reduced left ventricular systolic function and, most strongly, with in-hospital arrhythmic recurrence. Prospective multicenter studies are needed to clarify its prognostic value and assess whether improved guideline adherence can enhance patient outcomes.

## Figures and Tables

**Table 1:** Relevant medical history and clinical characteristics of the study population.

Variable	Study Population
Demographic characteristics:	
Age	71 (24–96)
Sex	84.47% (87)
BMI (Kg/m^2^)	26.0 (18.6–40.6)
Cardiovascular risk factors:	
Hypertension	58.16% (57)
Dyslipidemia	50% (49)
Diabetes mellitus type 2	12.24% (12)
Smoking	53.06% (52)
Active	30.77% (16)
Previous	69.23% (35)
Familiarity with CVD	27.55% (27)
Obesity (BMI > 30 Kg/m^2^)	22.45% (22)
Clinical history:	
Structural cardiopathy	90.20% (92)
LVEF (%)	35 (10–64)
AF/AFL	42% (42)
Previous VT	49.5% (50)
ICD	63.73% (65)
VT ablation	18.18% (18)
Cardiac denervation	2.02% (2)
Presenting symptoms:	
Chest pain	31.07% (32)
Dyspnea	16.5% (17)
Palpitations	25.24% (26)
Pre-syncope	14.56% (15)
Syncope	18.45% (19)
ICD shocks	25.24% (26)
Aspecific symptoms	21.36% (22)
Asymptomatic	10.68% (11)
Arrhythmia characteristics:	
Monomorphic	93.59% (73)
FC	166 (107–310)
QRS width (msec)	166 (100–400)
AV dissociation	17.46% (11)
Positive precordial concordance	10% (6)
Negative precordial concordance	13.33% (8)
Fusion and capture beats	4.48% (3)

**Table 2:** Distribution of antiarrhythmic strategy adopted during the whole hospitalization (ED + ward) to resolve sustained ventricular tachycardia.

Variable	Study Population
Amiodarone	50.49% (52)
Lidocaine	19.42% (20)
Verapamil	0%
Procainamide	0%
Magnesium	40.78% (42)
Catecholamines	1.94% (2)
Electrical cardioversion 1 line of treatment 2 lines of treatment	28.16% (29) 68.97% (20) 31.03% (9)
External defibrillation	1.94% (2)
Percutaneous stellate ganglion block	7.77% (8)

## Data Availability

The data that support the findings of this study are not publicly available but are available from the corresponding author upon reasonable request.

## Abbreviations

The following abbreviations are used in this manuscript:

VT	Ventricular Tachycardia
ED	Emergency Department
